# Maternal Vitamin D Status and the Relationship with Neonatal Anthropometric and Childhood Neurodevelopmental Outcomes: Results from the Seychelles Child Development Nutrition Study

**DOI:** 10.3390/nu9111235

**Published:** 2017-11-11

**Authors:** Eamon Laird, Sally W. Thurston, Edwin van Wijngaarden, Conrad F. Shamlaye, Gary J. Myers, Philip W. Davidson, Gene E. Watson, Emeir M. McSorley, Maria S. Mulhern, Alison J. Yeates, Mary Ward, Helene McNulty, J. J. Strain

**Affiliations:** 1 School of Medicine, Trinity College Dublin, Dublin 8, Ireland; lairdea@tcd.ie; 2 School of Medicine and Dentistry, University of Rochester, 265 Crittenden Blvd, Rochester, NY 14627, USA; Sally_Thurston@URMC.Rochester.edu (S.W.T.); Edwin_van_Wijngaarden@URMC.Rochester.edu (E.v.W.); Gary_Myers@URMC.Rochester.edu (G.J.M.); Phil_Davidson@URMC.Rochester.edu (P.W.D.); Gene_Watson@urmc.rochester.edu (G.E.W.); 3 The Ministry of Health, Mahé, Republic of Seychelles; shamlaye@gmail.com; 4 Nutrition Innovation Centre for Food and Health (NICHE), Ulster University, Cromore Road, Coleraine BT52 1SA, Northern Ireland, UK; em.mcsorley@ulster.ac.uk (E.M.M.); m.mulhern@ulster.ac.uk (M.S.M.); a.yeates@ulster.ac.uk (A.J.Y.); mw.ward@ulster.ac.uk (M.W.); h.mcnulty@ulster.ac.uk (H.M.)

**Keywords:** vitamin D, pregnancy, birth, childhood, neurodevelopment, health

## Abstract

Vitamin D has an important role in early life; however, the optimal vitamin D status during pregnancy is currently unclear. There have been recent calls for pregnant women to maintain circulating 25-hydroxyvitamin D (25(OH)D) concentrations >100 nmol/L for health, yet little is known about the long-term potential benefits or safety of achieving such high maternal 25(OH)D concentrations for infant or child health outcomes. We examined maternal vitamin D status and its associations with infant anthropometric and later childhood neurocognitive outcomes in a mother-child cohort in a sun-rich country near the equator (4.6° S). This study was conducted in pregnant mothers originally recruited to the Seychelles Child Development Nutrition Study. Blood samples (*n* = 202) taken at delivery were analysed for serum 25-hydroxyvitamin D (25(OH)D) concentrations. Multiple linear regression models assessed associations between maternal 25(OH)D and birth weight, infant head circumference, and neurocognitive outcomes in the children at age 5 years. Mothers were, on average, 27 years of age, and the children’s average gestational age was 39 weeks. None of the women reported any intake of vitamin D supplements. Maternal 25(OH)D concentrations had a mean of 101 (range 34–218 nmol/L) and none were deficient (<30 nmol/L). Maternal 25(OH)D concentrations were not associated with child anthropometric or neurodevelopmental outcomes. These findings appear to indicate that a higher vitamin D status is not a limiting factor for neonatal growth or neurocognitive development in the first 5 years of life. Larger studies with greater variability in vitamin D status are needed to further explore optimal cut-offs or non-linear associations (including for maternal health) that might exist among populations with sub-optimal exposure.

## 1. Introduction

Vitamin D plays a crucial role during pregnancy, owing to the potential to modulate foetal programming and the future health status of the offspring [1]. Neonates are heavily dependent on maternal vitamin D status, with severe deficiency resulting in poor skeletal mineralisation and increased risk of rickets [2], and insufficiency linked with a plethora of adverse neonatal outcomes including preterm birth, increased risk of infectious diseases and dental caries [3]. Maternal vitamin D status has been positively associated with birth weight [4,5,6,7] and bone mass of the child up to the age of nine years [8] in some observations, but not in all [9,10]. Again inconsistently, some studies [11,12,13], but not others [14], suggest that maternal vitamin D deficiency is also associated with adverse infant neurocognitive outcomes such as language impairment and autistic-like behaviours. Proposed mechanisms have included the presence of vitamin D receptors in the brain, and the influence of 1,25 dihydroxyvitamin D on the production of cytokines, plasticity and neurotransmission [15,16,17].

The primary source of vitamin D is exposure to UVB sunlight, which provides over 90% of daily requirements [18]. Age, body mass index (BMI), sunscreen use, clothing and latitude can all influence vitamin D synthesis, which is seasonal at high Northern and Southern latitudes owing to the variation in UVB intensity [19,20]. With seasonal synthesis, there is an increased reliance on dietary intakes during the winter period; however, few foods are rich in vitamin D, and these are infrequently consumed [21,22]. Not surprisingly, therefore, high rates of vitamin D deficiency and insufficiency have been reported during pregnancy at higher latitudes in countries such as Ireland, the U.K. and America [23,24,25]. Regardless of the lower cut-off points for deficiency, there have been recent calls for pregnant women to maintain circulating 25-hydroxyvitamin D (25(OH)D) concentrations >100 nmol/L for health [26]. However, little is known about the long-term potential benefits or safety of achieving such high maternal 25(OH)D concentrations for infant or child health outcomes. For instance, U- and J-shaped associations between 25(OH)D concentration and disease outcomes have been previously reported [27,28,29] and could also exist in relation to neurodevelopment and maternal vitamin D status. Adverse effects of elevated 25(OH)D concentration on both offspring behavior and physiology have been observed in animal models [30] but it is unknown on how translatable this is to humans.

Few studies have reported the vitamin D status during pregnancy in populations at equatorial regions where uninterrupted UVB exposure is hypothesised to provide optimal vitamin D synthesis throughout the year. Likewise, studies investigating the associations between vitamin D status and infant/child health outcomes have been limited to locations affected by seasonality, and therefore are not well placed to provide data on the influence of optimal vitamin D status. For instance, previous work has indicated that in developing countries birth weight can be affected by seasonal food shortages and seasonal fluctuations in infectious and parasitic diseases [31]. In other countries, influenza-related illness (which is often seasonal) during pregnancy can negatively influence gestational age and birth weight [32]. These confounding factors can make it difficult to separate and assess the individual impact of vitamin D.

Thus, the aims of this study were two-fold; firstly, to assess maternal delivery vitamin D status (25(OH)D) concentration) in a cohort of pregnant women living near the equator without sunshine seasonality; and secondly, to determine the associations between maternal vitamin D status and anthropometric measures at birth and neurocognitive development measures at age 5 years old.

## 2. Materials and Methods

### 2.1. Study Design

The population investigated in this study consisted of pregnant women (and their children) originally recruited to the Seychelles Child Development Nutrition Study (SCDNS) Nutrition Cohort 1 [33,34]. A total of 300 healthy pregnant women were recruited in 2001 during their first antenatal visit from nine antenatal clinics across Mahé, the main island of the Republic of Seychelles (located 4.7° south of the equator). The population are descendants of early French settlers and East Africans who arrived in the 19th century. The Tamil people, along with other South Indians and Chinese, account for the other permanent inhabitants of the country. The inclusion criteria for the original investigation included residency on Mahé, mothers aged over 16 years, and native-born Seychellois. Exclusion criteria included vegetarianism, chronic/serious diseases including preeclampsia with seizures, insulin-dependent diabetes, and haematological disorders such as sickle cell anaemia or thalassemia. Twenty-four women were excluded prior to or at birth, primarily because of terminated pregnancies, and a further 74 were not included because samples were not available for assessment of 25(OH)D concentrations. Data from 202 participants were available for this analysis; 172 participants were available for models of birth anthropometric measures and 177 for models of five-year outcomes after accounting for missing covariates and outcomes. All participants provided written informed consent and the study was conducted according to the guidelines in the Declaration of Helsinki. The study was originally reviewed and approved by the Seychelles Ethics Board and the Research Subjects Review Board at the University of Rochester in 2000 (RSRB# 8066). The study was re-approved by the Research Subjects Review Board at the University of Rochester on 2 March 2011 (RSRB# 35792).

### 2.2. Blood Collection and Laboratory Analysis

Pregnant women provided non-fasting blood samples (30 mL) that were collected by venepuncture into an evacuated tube by a trained phlebotomist one day after delivery [35]. Samples were placed on water ice and allowed to sit for 30 min prior to being centrifuged (1000× *g* for 15 min) within 3 h of collection, with serum aliquots stored at −80 °C until analysis. For the current investigation, stored samples were accessed for vitamin D analysis; total serum 25(OH)D (D2 + D3) concentrations were quantified by a fully validated method (Chromsystems Instruments & Chemicals GmbH, Munich, Germany (MassChrom^®^ 25-OH-Vitamin D3/D2), using liquid chromatography-tandem mass spectrometry (API 4000, AB SCIEX, Foster City, CA, USA), and batch analysed at Ulster University. The quality and accuracy of the method was monitored using internal quality controls and by participation in the vitamin D External Quality Assessment Scheme (DEQAS) and use of the National Institute of Standards and Technology (NIST) 972 vitamin D standard reference material. The respective inter- and intra-assay CVs were 5.7% and 4.5%. For this study, vitamin D sufficiency was defined as a serum 25(OH)D concentration ≥50 nmol/L, insufficiency as 30–49.9 nmol/L, and deficiency as <30 nmol/L [36].

### 2.3. Maternal Demographic and Lifestyle Assessment

Demographic and behavioural data were obtained from questionnaires and included: maternal age, smoking history, fish intake (which was part of a 4-day food diary (two consecutive weekdays and two weekend days)), alcohol intake, parity and socio-economic status (Hollingshead SES score) [26,27]. Mothers completed the Kaufman Brief Intelligence Test (Matrices (KBIT-M)), and study nurses completed the Paediatric Review of Children’s Environmental Support and Stimulation (PROCESS). The Seychelles population is a high fish-eating population, and fish is the primary source of exposure to methylmercury (MeHg), a neurotoxicant at sufficient dosage. Measures of maternal hair MeHg were available from pregnancy hair samples collected at delivery and fatty acid concentrations were measured previously from maternal blood taken at 28 weeks and at delivery [26,27]. BMI (kg/m^2^) was calculated based on height to the nearest 0.01 m (using a stadiometer), and weight to the nearest 0.01 kg (using electronic scales). Measuring equipment was calibrated prior to and throughout the duration of the study by the Seychelles Bureau of Standards.

### 2.4. Infant and Child Health Measures

Birth outcome data, including birth weight and head circumference, were obtained from hospital records. When the children were nearing the age of 5 years, they were recalled for an evaluation. Neurocognitive tests conducted on the children included finger tapping ((FT) dominant and non-dominant hands); three subtests of the Preschool Language Scale-Revised Edition (Total Language Score (PLS-TL), Verbal Ability Score (PLS-VA), and Auditory Comprehension (PLS-AC)); two subtests of the Woodcock-Johnson Scholastic Achievement Test, second edition (Letter Word Identification and Applied Problems); the Child Behaviour Checklist (CBCL) total t score; and two subtests of the Kaufman Brief Intelligence Test (Verbal Knowledge (KBIT-VK) and Matrices (KBIT-M)). In general, higher scores indicate improved performance, except for FT and CBCL [37].

### 2.5. Statistical Analysis

The cohort characteristics were examined in descriptive analyses, including means, standard deviations and ranges. Because fish intake may be a source of vitamin D in addition to UVB exposure, we assessed the correlation between 25(OH)D and both maternal fish consumption and MeHg exposure, with and without using the logarithmic transformation of 25(OH)D concentration due to the non-normal distribution. Both unadjusted linear regression, and minimally and fully adjusted multivariable linear regression models were utilized to investigate associations between 25(OH)D and birth weight and head circumference, and neurocognitive tests at five years of age. As per previous reports [34,37], covariates were included in the fully adjusted models if they were identified a priori as potential confounders or predictors of birth outcomes or neurodevelopment. Covariates were included in the minimally adjusted model if they were associated with vitamin D status and any outcome of interest (*p* < 0.1) and therefore potential confounders, or if they were only significant predictors of any birth or neurodevelopmental outcome (*p* < 0.05) and therefore improve model fit. These evaluations were made separately for birth outcomes and neurodevelopmental outcomes. None of the covariates were considered potential mediators or colliders. The number of observations were kept the same across all three types of models such that the impact of adding model covariates on vitamin D associations could be assessed. Only maternal BMI at enrolment was significantly associated with a birth outcome (head circumference) and vitamin D status, and was therefore the only potential confounder in birth outcomes models (no potential confounders were identified for neurodevelopmental outcomes). All other covariates in the minimally adjusted models were significant predictors for one or more outcomes. For birth outcomes, minimally adjusted models included child’s sex, gestational age, and maternal BMI at enrolment. Fully adjusted models included maternal MeHg, child’s sex, alcohol and tobacco use in pregnancy, diabetes, gestational age, maternal age, delivery weight gain, maternal BMI at enrolment, mean DHA and mean AA concentrations, Hollingshead SES, and number of other living children. For neurocognitive outcomes, minimally adjusted models included child’s sex, child age at testing, maternal intelligence (assessed by KBIT-M), Hollingshead SES, and the PROCESS. Fully adjusted models included maternal MeHg, child’s sex, child age at testing, family status at 5 years (1 if living with both parents, 0 if not), maternal age, birth weight, mean DHA and mean AA concentrations, maternal intelligence, Hollingshead SES, and the PROCESS.

All model covariates were treated as continuous variables unless otherwise noted. All outcomes are continuous variables, for which linear regression is appropriate. Model assumptions were checked using standard methods including residual plots to check for constant variance, linearity, and normality, and variance inflation factors to check for multicollinearity; all model assumptions were reasonably well met. Statistical analyses were performed using R (version 3.0.2; The R Foundation for Statistical Computing). Power calculations were based on results from Mannion et al. who report relationships between vitamin D intake from repeat 24-hour dietary diaries during pregnancy and infant birthweight [5]. All power calculations use alpha= 0.05 and assume a 2-sided test. Based on the reported slope from the regression of birthweight on vitamin D, as well as the SD of both vitamin D and birth weight, the correlation between vitamin D and birth weight was approximately 0.30 in the Mannion et al. study. If the correlation between 25(OH)D concentrations and birth weight in our SCDNS cohort was this large, we would have 98% power to detect a significant relationship between these variables with the n=172 subjects available for this analysis; we would have 80% power if the correlation was 0.22. Power was calculated using the pwr.r.test function in the pwr package in R.

## 3. Results

The baseline characteristics of both the mothers and neonates are presented in Table 1 and Table 2. The mean age of the mothers was 27.5 years with a low percentage of smokers during pregnancy (3%) while 46% reported alcohol intake (Table 1). The average gestational age was 38.6 weeks with a mean birth weight of 3242 g (Table 2). None of the women reported taking vitamin D supplements while the neurodevelopmental test outcome scores were all within the normal ranges for this age group. The mean (range) 25(OH)D concentration at delivery was 101.2 nmol/L (34-218 nmol/L) and the proportion of those classified as vitamin D sufficient (25(OH)D > 50 nmol/L) was 98%. Assuming a higher cut-off of 75 nmol/L for vitamin D sufficiency (suggested as an alternative by some authors [38]), this would decrease to 86%.

Vitamin D status was not associated with either maternal MeHg, or maternal fish consumption, either using untransformed or log transformed 25(OH)D concentrations (data not shown). The associations with neonatal birth weight and head circumference and neurodevelopmental outcomes are summarized in Table 3, where β indicates the change in outcome per nmol/L change in 25(OH)D. Maternal 25(OH)D concentration at delivery was not a significant predictor of either birth weight or head circumference in either unadjusted or covariate-adjusted models. Concentrations of 25(OH)D at delivery were not associated with any neurocognitive development outcome measure at 5 years of age in unadjusted or adjusted models (Table 3).

## 4. Discussion

In this observational study of pregnant women located near the equator, over 98% had sufficient vitamin D status (>50 nmol/L) at delivery, which was as expected, given their year-round UVB exposure and uniquely high fish consumption. However, we observed no associations (positive or adverse) of maternal 25(OH)D concentrations (up to 218 nmol/L) with the infant’s birth weight, head circumference, or their neurocognitive outcomes at 5 years of age.

Our findings of no association of vitamin D with neurodevelopment is supported by a number of other studies. Darling et al. reported maternal 25(OH)D (interquartile range: 45.2–90.4 nmol/L) was not associated with neurodevelopment outcomes (including IQ or reading ability) in older children aged >4–9 years old in a cohort of 7065 mother-child pairs from southwest England [39]. Additionally, maternal 25(OH)D (5th to 95th percentiles: 23–152 nmol/L) was not associated with scholastic achievement in Danish schoolchildren (*n* 850) after 10 years of schooling [40] while Gale et al. observed no associations between maternal 25(OH)D status >75 nmol/L with measures of cognitive development in children from the United Kingdom at age 9 years [41]. However, these results are in contrast to Morales et al., who reported a positive linear relationship in a Spanish cohort between maternal vitamin D status and mental and psychomotor skills in infants (*n* 1820; maternal 25(OH)D interquartile range: 54.4–93.1 nmol/L) [11], while Whitehouse et al. reported a twofold risk of having a child with language difficulties in Australian women with maternal vitamin D insufficiency (≤46 nmol/L) in comparison with mothers with 25(OH)D concentration >70 nmol/L [12].

Studies of vitamin D and anthropometric outcomes are more consistent. In the current study, we observed no associations of maternal 25(OH)D with any birth outcome. This is similar to Ong et al., where maternal 25(OH)D (mean 81.3 nmol/L) was not associated with infant growth or adiposity outcomes in >700 infants based in Singapore [42]. Morales et al. reported no association of maternal 25(OH)D (median 74 nmol/L) with birth length or weight in >2000 Spanish infants [43], while in 559 English infants, maternal 25(OH)D (median 37.8 nmol/L) was unrelated to newborn anthropometry outcomes [10]. Similar results have been observed in other studies [9,41], while a Cochrane review showed that supplementation with vitamin D in pregnancy did not affect birth length or weight [44]. However, RCTs have demonstrated some positive effects of maternal supplementation on infant head circumference, though the supplement doses varied widely from 200 IU [45] per day to 400 IU daily (plus 50,000 IU for 8 weeks) [46] or 60–120,000 IU doses [47].

In explaining the null findings in our study and others, the argument could be made that the failure to detect significance was owing to the low prevalence of inadequate status in our cohort (~2%) and that associations are only evident in populations with lower vitamin D status. While it is true that some studies with a relatively high mean 25(OH)D (>50 nmol/L) failed to observe any significance with either infant growth [9,42] or scholastic outcomes [39,40], it is important to note that other studies with lower 25(OH)D status also failed to show associations [10]. It is possible that that once a certain 25(OH)D concentration has been reached (~50 nmol/L) at a specific pregnancy time-point, higher concentrations have no further effect on anthropometric or neurocognitive measures until one reaches toxicity, which can then cause hypercalcemia, nausea and weakness. However, this hypothesis needs verification with future studies in populations with greater variability in vitamin D status. Such studies could also examine U- and J-shaped associations, which have been reported between 25(OH)D concentration and disease outcomes [27,28,29], and have been suggested to exist in relation to child neurodevelopment and maternal vitamin D status. However, in the current analysis, we observed no adverse associations of maternal 25(OH)D concentrations up to 218 nmol/L with the children’s outcomes and all neurocognitive test scores were within the normal range for this age group. Indeed none of the other studies reported any adverse anthropometric or neurocognitive outcomes from elevated maternal 25(OH)D concentrations (>50 nmol/L) potentially suggesting supplementation to achieve up to 200 nmol/L is safe in relation to these specific health outcomes.

This potential safety for infant and child outcomes is important, given that high maternal 25(OH)D concentrations could be protective against preeclampsia [25], a serious condition which is one of the commonest causes of maternal morbidity and mortality [48]. Furthermore, there may be additional maternal health benefits, particularly for those located at high latitudes, as it has been suggested that the seasonal cycling of vitamin D may have negative perturbations for the activity of various vitamin D related hydroxylase enzymes, which could result in an increased risk of adverse health outcomes [49,50]. Additionally, a recent meta-analysis from 25 randomised controlled trials found that vitamin D supplementation protected against acute respiratory tract infections [51], which, in pregnancy, could increase the placental transfer of maternal antibodies and disrupt foetal neurodevelopment by cross-reacting with foetal brain antigens [52,53]. However, future studies are needed to examine these outcomes.

In terms of what vitamin D status is achievable in pregnancy, both O’Riordan et al. and Holmes et al. reported high levels of vitamin D insufficiency (25–50 nmol/L) and deficiency (<25 nmol/L) in Caucasian pregnant women located in Ireland [24] and the UK [23] (51–55° N), while countries often viewed as ‘sun-rich’, such as Greece, have also reported a high prevalence of deficiency (<25 nmol/L) within the pregnant population [54], partly owing to seasonal variation in UVB sun exposure. The only study that reported comparable/higher 25(OH)D concentrations than the current study was a cohort of indigenous Sengrema, Same and Ukerewe pregnant women (*n* 139) from Tanzania (2–5° S), with a mean 25(OH)D concentration of 138.5 nmol/L [55]. In other countries closer to the equator, vitamin D inadequacy during pregnancy is still reported. Among samples of pregnant women from Hanoi City and rural Hai Duong Province, Vietnam (15° N; *n* = 541), and from Thailand (14° N; *n* = 120) 48% and 47.2% were deemed insufficient, respectively, using a cut-off of <75 nmol/L [56,57]. More recently, in a Brazilian cohort of 229 pregnant women (23°S), the prevalence of 25(OH)D <75 nmo/L was 33.9% [58], while 13.2% of 910 pregnant women from Singapore (1°22′ N) had maternal concentrations <50 nmol/L [42]. Indeed, even within the current study, a degree of insufficiency was observed when using the 75 nmol/L cut-off. These findings suggest that other potential factors, such as clothing, vitamin supplementation, diet, socio-economic factors, physiology and obesity may also influence the ability to achieve presumed optimal status [19,20]. For example, although 25(OH)D was not correlated with fish intake, the majority of the Seychelles population consume large quantities of fish as part of their normal diet [59], which could have significantly contributed to the already high 25(OH)D concentration obtained from sunshine exposure [60]. Recently, the American Institute of Medicine (IOM) updated the recommended dietary intake for pregnant women of vitamin D from 5 µg to 15 µg/d, while in the UK and Ireland, the reference nutrient intake is set at 10 µg/d [36,61,62]. It has been estimated that a dietary intake of 41.1 µg/d of vitamin D is required to maintain a continual 25(OH)D concentration >80 nmol/L throughout the year (within the general population), which is approaching (but still below) the mean concentration observed in the current study [63]. However, recent studies indicate that actual dietary intakes of pregnant women are significantly below these intake levels [64,65]. Thus, to achieve the 25(OH)D concentrations observed in the current study, mandatory food fortification of foods or increased vitamin D supplementation would be necessary.

This study has several strengths. To the best of our knowledge, this is the first study to measure maternal vitamin D status and estimate its association with child anthropometric and neurocognitive measures (up to the age of 5 years) in a population located in an equatorial region with unvarying UVB sun exposure. Vitamin D status was also assessed using LC-MS/MS, which is increasingly becoming the gold standard of vitamin D assessment [36]. Furthermore, we were able to account for a number of covariates that are known to influence birth and developmental outcomes. Our study also has some limitations. In particular, the small overall sample size and lack of variability of vitamin D status within the insufficient range did not allow us to identify a vitamin D cut-off level that could be considered a minimum threshold for optimal foetal and child development. Larger studies with greater variability in vitamin D status, especially in the tail ends of the distribution, are needed for more detailed exploration of an optimal cut-off or non-linear associations. Furthermore, we also had no data available in relation to sun-exposure or dietary vitamin D intakes although at this location UVB exposure is fairly uniform and we were able to adjust for fish intakes. It is also important to note that the comparison of maternal 25(OH)D with birth outcomes was a cross-sectional analysis and longitudinal measurements of 25(OH)D during pregnancy may provide more data to explore potential associations.

## 5. Conclusions

In conclusion, we observed nearly universal vitamin D sufficiency among a cohort of mothers living near the equator, reflecting the 25(OH)D concentrations which are achievable during pregnancy without seasonality. At relatively high, un-supplemented 25(OH)D concentrations, we did not observe any associations with birth outcomes or neurodevelopmental tests administered to the offspring at age 5 years. These findings appear to indicate that having a high vitamin D status is not a limiting factor for neonatal growth or neurocognitive development in the first 5 years of life. This lack of any observations of adverse effects on infant or child growth could be advantageous for pregnant women who maintain higher 25(OH)D concentrations for maternal health. However, further research is needed to identify either any currently undetected adverse health effects of attaining such high 25(OH)D maternal concentrations and also the potential wider health benefits for both maternal and child health.

## Figures and Tables

**Table 1:** Maternal mean, SD, and range for demographic and 25(OH)D measures ^1^.

Variable	*n*	Mean	SD	Min	Max
Delivery 25(OH)D (nmol/L)	202	101.20	27.30	34.00	218.00
Maternal BMI at enrolment	201	26.38	6.51	16.06	50.03
Delivery weight gain (kg)	201	9.69	4.72	0.10	23.20
Diabetes (%)	202	3			
Alcohol in pregnancy (%)	202	46			
Tobacco use in pregnancy (%)	201	3			
Fish meals (per 2 weeks)	199	17.75	8.13	3.00	58.00
Maternal hair mercury (ppm)	202	5.42	3.70	0.44	22.71
Maternal serum DHA (mg/mL)	199	0.03	0.01	0.01	0.05
Maternal serum AA (mg/mL)	199	0.10	0.02	0.04	0.16
Number other living children	186	1.27	1.26	0.00	5.00
Maternal KBIT	191	84.44	14.25	48.00	117.00
Hollingshead SES (9 months)	194	32.96	11.06	14.00	60.00
Hollingshead SES (5 years)	189	30.51	10.98	8.00	58.50

^1^ Non-excluded participants who have measures of 25(OH)D and at least one outcome. Abbreviations: Min, minimum; Max, maximum; 25(OH)D, 25-hydroxyvitamin D; KBIT, Kaufman Brief Intelligence test; DHA, docosahexaenoic acid; AA, arachidonic acid; BMI, body mass index; SES, socio-economic status.

**Table 2:** Child mean, SD, and range for demographic, birth outcome and neurocognitive measures.

Variable	*n*	Mean	SD	Min	Max
Girl (%)	202	49			
Gestational age (week)	201	38.67	1.35	34.00	41.00
Birth weight (gm)	201	3242.70	504.40	1654.00	4450.00
Head circumference (cm)	201	33.56	1.37	30.00	37.40
**At age 5 years:**					
Child age at 5-year test	189	5.54	0.28	5.14	6.32
Family status (living with both parents 5 years,%)	189	41			
FT dominant hand	189	23.56	5.41	8.40	37.40
FT non-dominant hand	189	21.40	4.84	9.00	34.40
PLS total language	189	118.03	5.44	100.00	128.00
PLS auditory	189	55.27	2.73	47.00	60.00
PLS verbal	189	62.76	3.30	51.00	68.00
WJ applied problems	189	14.42	3.96	2.00	23.00
WJ letter word	189	9.80	5.71	1.00	24.00
CBCL total t score	189	59.73	8.67	25.00	77.00
KBIT verbal	189	11.44	2.76	6.00	17.00
KBIT matrices	189	7.63	1.24	2.00	9.00
PROCESS	186	151.59	14.98	116.00	190.00

Abbreviations: Min, minimum; Max, maximum; FT, finger tapping; PLS, Pre-school language score; WJ, Woodcock-Johnston Scholastic Achievement test; CBCL, Child behavior checklist; KBIT, Kaufman Brief Intelligence test; PROCESS, Pediatric Review of Children’s Environmental Support and Stimulation. In general, higher scores for the cognitive tests indicate improved performance, except for FT and CBCL.

**Table 3:** Slopes (β) and 95% confidence intervals (95% CI) relating 25(OH)D concentrations at delivery to birth outcomes and neurocognitive outcomes at 5 years of age ^1^.

	Unadjusted		Minimally adjusted		Fully adjusted	
	β	95% CI	β	95% CI	β	95% CI
Birthweight (gm)	2.149	(−0.656, 4.954)	2.067	(−0.452, 4.586)	2.125	(−0.472, 4.722)
Head circumference (cm)	0.003	(−0.005, 0.011)	0.004	(−0.004, 0.012)	0.004	(−0.004, 0.012)
FT dominant hand	0.017	(−0.011, 0.045)	0.015	(−0.013, 0.043)	0.018	(−0.01, 0.046)
FT nondominant hand	0.005	(−0.021, 0.031)	0.004	(−0.022, 0.03)	0.005	(−0.023, 0.033)
PLS total language	0.01	(−0.02, 0.04)	0.014	(−0.014, 0.042)	0.018	(−0.01, 0.046)
PLS auditory	0.006	(−0.01, 0.022)	0.007	(−0.007, 0.021)	0.008	(−0.006, 0.022)
PLS verbal	0.004	(−0.014, 0.022)	0.006	(−0.012, 0.024)	0.009	(−0.009, 0.027)
WJ applied problems	0.001	(−0.021, 0.023)	0.003	(−0.017, 0.023)	0.005	(−0.015, 0.025)
WJ letter word	−0.008	(−0.04, 0.024)	−0.005	(−0.029, 0.019)	−0.007	(−0.031, 0.017)
CBCL total t score	−0.002	(−0.049, 0.045)	−0.007	(−0.052, 0.038)	−0.011	(−0.058, 0.036)
KBIT verbal	−0.011	(−0.027, 0.005)	−0.01	(−0.024, 0.004)	−0.009	(−0.023, 0.005)
KBIT matrices	−0.002	(−0.008, 0.004)	−0.002	(−0.008, 0.004)	−0.003	(−0.009, 0.003)

^1^ From separate linear regression models. Birth outcomes (birth weight, head circumference): minimally adjusted models included child’s sex, gestational age, and maternal BMI at enrolment; fully adjusted models included maternal MeHg, child’s sex, alcohol and tobacco use in pregnancy, diabetes, gestational age, maternal age, delivery weight gain, maternal BMI at enrolment, mean DHA and mean AA concentrations, socioeconomic status (SES) and number of other living children. Neurocognitive outcomes: minimally adjusted models included child’s sex, child age at testing, maternal intelligence (assessed by KBIT-M), socioeconomic status (SES) and the PROCESS; fully adjusted models included maternal MeHg, child’s sex, child age at testing, family status at 5 years (1 if living with both parents, 0 if not), maternal age, birth weight, mean DHA and mean AA concentrations, maternal intelligence (assessed by KBIT-M), socioeconomic status (SES) and the PROCESS.

## Abbreviations

AA	Arachidonic acid
AC	Auditory Comprehension
CBCL	Child behavior checklist
DHA	Docosahexaenoic acid
FT	Finger tapping
KBIT	Kaufman Brief Intelligence test
MeHg	Methylmercury
PLS	Pre-school language score
PROCESS	Pediatric Review of Children’s Environmental Support and Stimulation
SES	Socio-economic status
TL	Total Language Score
VA	Verbal Ability Score
WJ	Woodcock-Johnston Scholastic Achievement test
25(OH)D	25-hydroxyvitamin D
